# Elevated Muscle-Specific miRNAs in Serum of Myotonic Dystrophy Patients Relate to Muscle Disease Progress

**DOI:** 10.1371/journal.pone.0125341

**Published:** 2015-04-27

**Authors:** Andrie Koutsoulidou, Tassos C. Kyriakides, George K. Papadimas, Yiolanda Christou, Evangelia Kararizou, Eleni Zamba Papanicolaou, Leonidas A. Phylactou

**Affiliations:** 1 Department of Molecular Genetics, Function & Therapy, Cyprus Institute of Neurology & Genetics, P.O. Box 2346, 1683 Nicosia, Cyprus; 2 Yale Center for Analytical Sciences,Yale School of Public Health, 300 George Street, Suite 555, New Haven, CT 06520, United States of America; 3 Department of Neurology, Eginitio hospital, Medical School of Athens, 74 Vasilissis Sofias, 11528, Athens, Greece; 4 Neurology Clinic D, Cyprus Institute of Neurology & Genetics, P.O. Box 2346, 1683 Nicosia, Cyprus; University of Texas MD Anderson Cancer Center, UNITED STATES

## Abstract

The discovery of reliable and sensitive blood biomarkers is useful for the diagnosis, monitoring and potential future therapy of diseases. Recently, microRNAs (miRNAs) have been identified in blood circulation and might have the potential to be used as biomarkers for several diseases and clinical conditions. Myotonic Dystrophy type 1 (DM1) is the most common form of adult-onset muscular dystrophy primarily characterized by muscle myotonia, weakness and atrophy. Previous studies have shown an association between miRNAs and DM1 in muscle tissue and, recently, in plasma. The aim of this study was to detect and assess muscle-specific miRNAs as potential biomarkers of DM1 muscle wasting, an important parameter in the disease’s natural history. Disease stable or progressive DM1 patients with muscle weakness and wasting were recruited and enrolled in the study. RNA isolated from participants’ serum was used to assess miRNA levels. Results suggest that the levels of muscle-specific miRNAs are correlated with the progression of muscle wasting and weakness observed in the DM1 patients. Specifically, miR-1, miR-133a, miR133b and miR-206 serum levels were found elevated in DM1 patients with progressive muscle wasting compared to disease stable DM1 patients. Based on these results, we propose that muscle-specific miRNAs might be useful molecular biomarkers for monitoring the progress of muscle atrophy in DM1 patients.

## Introduction

Myotonic dystrophy type 1 (DM1) is the most common form of adult-onset muscular dystrophy. Clinically, it is a highly variable multisystemic disorder inherited in an autosomal dominant manner [1]. The symptoms and severity of DM1, ranges from mild to severe, and frequently result in death due to respiratory deficiency [2]. DM1 is caused by a trinucleotide CTG repeat expansion located in the 3′ untranslated region (3′UTR) of the *dystrophia myotonica protein kinase* (*DMPK*) gene on chromosome 19ql3.3 [3–6]. The number of CTG repeats ranges between five and thirty-five in the normal population and increases to between fifty and several thousand in DM1 patients. Longer alleles are associated with a more severe form of the disease and an earlier age of onset [7–9]. Expanded disease-associated alleles are highly unstable in individuals with DM1 in both somatic and germ lines [10]. Instability in germ lines is strongly biased towards expansion, providing a molecular explanation for the increase in severity through generations (genetic anticipation) [11, 12]. Somatic instability is size- and age-dependent and tissue-specific causing compromised genotype-phenotype correlations [13–15]. It has been shown that the CTG repeat instability is caused by replication errors or processes independent of cell division such as exogenously added compounds and environmental factors [16–21]. This instability leads to somatic mosaicism for the size of the CTG repeat expansion [10, 11]. The correlation between CTG repeat size observed in one tissue (e.g. blood) often does not match the severity of the disease and the CTG repeat size in other organs (e.g. muscle) [16]. Furthermore, repeat size is often stable in some postnatal tissues (e.g. blood) but not in others (e.g. skeletal and cardiac muscle) [16]. The CTG repeat expansion heterogeneity was shown to be tissue-specific, with heart and skeletal muscle DNA having the largest expansions most frequently [22]. Correlation of CTG repeat size measured in blood with specific symptoms is poor or even undetectable [23–25]. Furthermore, the CTG repeat size in muscle provides an even worse genotype-phenotype correlation [26]. Therefore, it is possible that allele length is not the major modifier of either disease severity or age of onset [27]. Above the lower disease-causing threshold of repeats, there is another upper threshold beyond which an increasing repeat length makes no additional contribution toward age at onset; this implies the poor genotype-phenotype correlations observed in DM1 [28, 29].

The primary characteristics of DM1 are myotonia, muscle weakness and atrophy. A very common symptom in DM1 patients is weakness of distal muscles of the extremities which later spreads to the proximal muscles [30]. In addition, there is early involvement of the facial muscles [30]. Muscle wasting has not been correlated with CTG repeat expansions or any other molecular biomarker, and is being currently monitored mainly through physical and clinical examination [16].

Many scientific reports emphasize the need for the creation of serum-based diagnostic methods, since they are easily accessible and convenient [31, 32]. An ideal serum biomarker of a muscular condition should be abundant, preferentially or exclusively produced in muscle tissue, resistant to degradation from enzymes that exist in blood circulation and present at low concentrations in the bloodstream of healthy people. Recently, microRNAs (miRNAs), small non-coding regulatory RNA molecules, have been identified to be present at significant levels in extracellular body fluids, including blood serum and plasma. It has thus been suggested that they can be used as potential biomarkers for different situations [33–36]. Further characterization of the serum miRNA expression profiles under normal physiological conditions and in different disease states have shown that the miRNAs present in serum are derived not only from circulating blood cells but also from other tissues/organs directly affected by disease [37–41]. The unique expression patterns of these serum miRNAs have therefore the potential to be used as clinical non-invasive biomarkers for diagnosis of various diseases. Recent studies suggest that muscle-specific miRNAs are present in the serum of animal models for Duchenne Muscular Dystrophy (DMD), supporting the idea that serum miRNAs are useful and reliable biomarkers for muscular dystrophies [42]. Additional studies indicate that muscle-specific miRNAs are present in blood circulation and have the potential to be used as diagnostic tools since their levels were determined to correlate with the severity of DMD disease [43]. Muscle-specific miRNAs have been shown to be altered in muscle biopsies of DM1 patients [44–46]. Very recently, Perfetti et al identified a signature of nine deregulated miRNAs in plasma samples of DM1 patients and suggested that these miRNAs can be used as diagnostic biomarkers for DM1 [47]. The muscle-specific miR-133a was included in the nine deregulated miRNAs in plasma samples of DM1 patients [47].

The most important symptom indicating DM1 disease progression is muscle atrophy. The aim of this study was to detect and assess potential biomarkers for monitoring muscle weakness and progressive atrophy in DM1 patients. In the present work, we show that muscle-specific miRNAs are elevated in the blood circulation of DM1 patients. Notably, the serum levels of miR-1, miR-133a, miR-133b and miR-206 appear to be correlated with progression of muscle wasting in DM1 patients. These results indicate that muscle-specific miRNAs may be useful tools for monitoring the progress of DM1 muscle wasting.

## Materials and Methods

### Participant inclusion, blood collection and isolation of serum

The study was approved by the National Bioethics Committee of Cyprus and participants provided a written informed consent to participate and provide blood specimens to the study. All DM1 patients were previously diagnosed by (a) the Diagnostic Department, Cyprus Institute of Neurology and Genetics using Southern blot technique and (b) the Diagnostic Department, Eginitio Hospital using Long-PCR and Fragment Analysis and TP-PCR methods. For the purposes of the project all DM1 patients were physically examined prior to study enrolment. None of the participants were taking steroids or diabetes treatment (such medicines may affect the levels of circulating miRNAs). Healthy participants completed a health-status questionnaire in order to verify they were free of any serious medical history or recent illness (more than a year) and were not being treated for a chronic medical condition (S1 and S2 Tables). The healthy participants did not have a family history of muscle disease. Following clinical examination, a total of 4 ml of blood was drawn from all study participants and placed in plain serum collection tubes (BD Vacutainer, U.S.A.). For DM1 patients, blood collection for miRNA analysis was performed following their last clinical examination. Serum was subsequently isolated from the samples.

### miRNA isolation and analysis

Following serum collection, total RNA, including miRNAs, was extracted from serum samples using the mirVana PARIS Kit (Applied Biosystems), according to the manufacturer’s instructions. A total of 10 ng of the extracted RNA was subjected to Reverse Transcriptase PCR using the TaqMan MicroRNA Reverse Transcription Kit (Applied Biosystems), according to the manufacturer’s instructions. Real-Time PCR amplification was performed using TaqMan MicroRNA Assays to measure miRNAs levels. miRNA detection assays specific for miR-1, miR-133a, miR-133b and miR-206 (Applied Biosystems) were carried out according to the manufacturer’s instructions. miRNA expression was normalized to the miR-16 (Applied Biosystems). Data analysis was performed using the SDS 2.4 Real-Time PCR data analysis software (Applied Biosystems).

### Statistical analysis

Statistical analysis was performed as described before [48]. ΔCt values were calculated from Ct (miR-16) minus Ct (miRNA). Normality of the distribution of each of the miRNA variables was assessed using the Shapiro-Wilk test; non-parametric methods (exact Wilcoxon tests) were used in the analyses. A two-tailed p-value of 0.05 was used to determine statistical significance. A Bonferroni adjustment was made to the alpha-level (from 0.05 to 0.00185) to account for multiple comparisons (n = 27). Spearman’s correlation analyses were carried out to assess correlations between miRNA levels and study participant demographic, clinical and molecular characteristics. Differences between DM1 patients and healthy participants were assessed using chi-square (categorical variables) and Wilcoxon tests (continuous variables). In addition, receiver operating characteristic (ROC) curves were used to determine the sensitivity and specificity of the assays in discriminating between (a) DM1 patients and healthy participants and (b) progressive vs non-progressive DM1 patients. The area under the curve (AUC) for the ROC curves was calculated. All analyses were performed using SAS, v.9.3 (SAS Institute Inc., Cary, NC, USA) software.

## Results

### miR-1, miR-133a, miR-133b and miR-206 levels are elevated in the blood serum of DM1 patients

Despite the fact that DM1 is considered to be a multi-systemic disorder, muscle wasting and weakness is considered to be a very important and serious characteristic of DM1 patients. In order to assess potential molecular biomarkers for monitoring muscle wasting and weakness in DM1 patients, we investigated miRNA levels that are specifically expressed in muscle tissue; this would allow for characterisation of the progression of muscle wasting independent of other tissues that might possibly be affected in DM1 patients. The four muscle-specific miRNAs, miR-1, miR-133a, miR-133b and miR-206, were previously shown to exist in blood circulation of patients with DMD [43].

**Fig 1:**
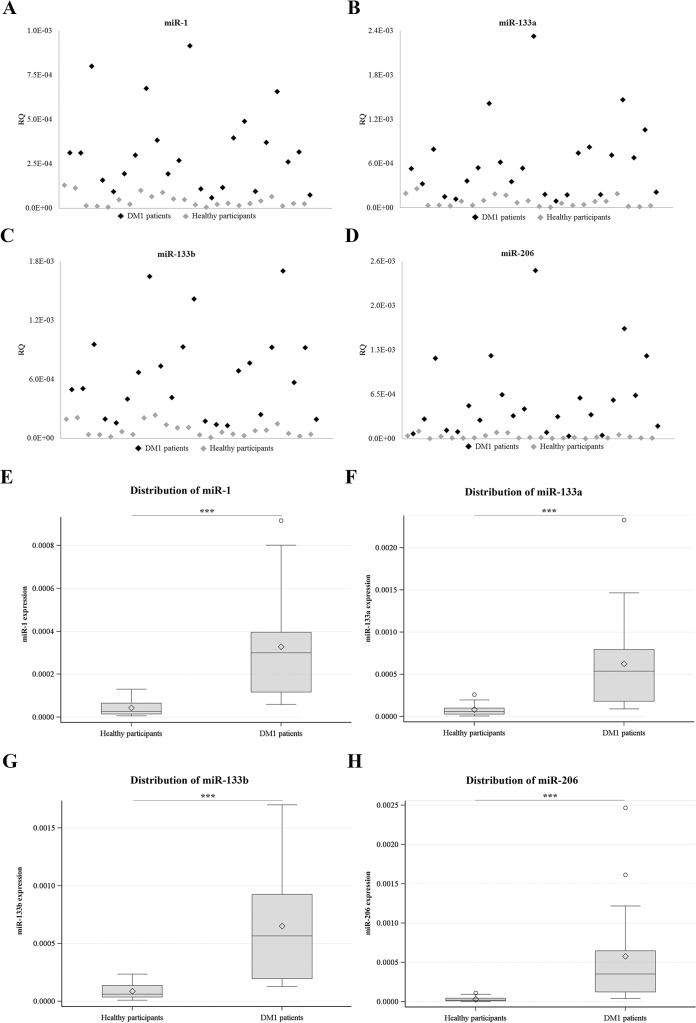
Muscle-specific miRNAs levels are elevated in the serum of DM1 patients. Serum samples from twenty three DM1 patients and twenty three healthy participants were analysed for the presence of muscle-specific miRNAs. miR-1 (**A**), miR-133a (**B**), miR-133b (**C**) and miR-206 (**D**) were significantly elevated in the serum of DM1 patients compared to the serum of healthy participants (p<0.005). Distribution charts of miR-1 (**E**), miR-133a (**F**), miR-133b (**G**) and miR-206 (**H**) levels in DM1 patients and healthy participants show the elevated levels of the muscle-specific miRNAs in the serum of DM1 patients compared to healthy participants. Horizontal lines inside the boxes mark the medians. Mean expression values are marked with rhombus. *** p < 0.0001.

**Fig 2:**
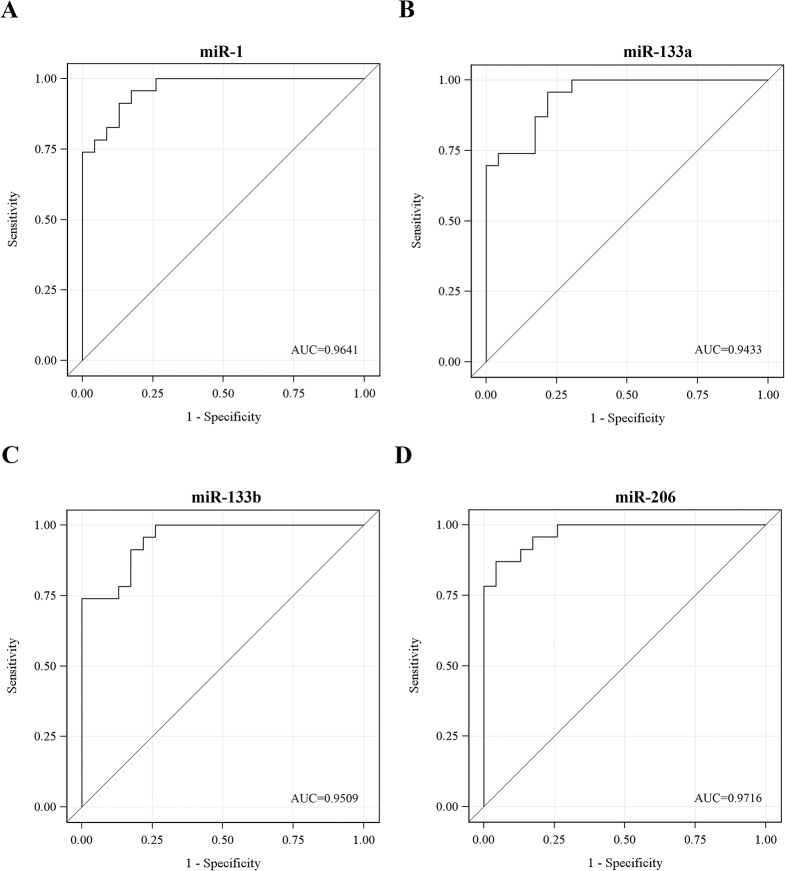
Receiver-operator characteristics (ROC) curve analyses of muscle-specific miRNAs discriminates DM1 patients from healthy participants. ROC curve analyses using serum miR-1 (**A**), miR-133a (**B**), miR-133b (**C**) and miR-206 (**D**) for discriminating healthy participants from DM1 patients. Area under the curve (AUC) values are presented.

**Fig 3:**
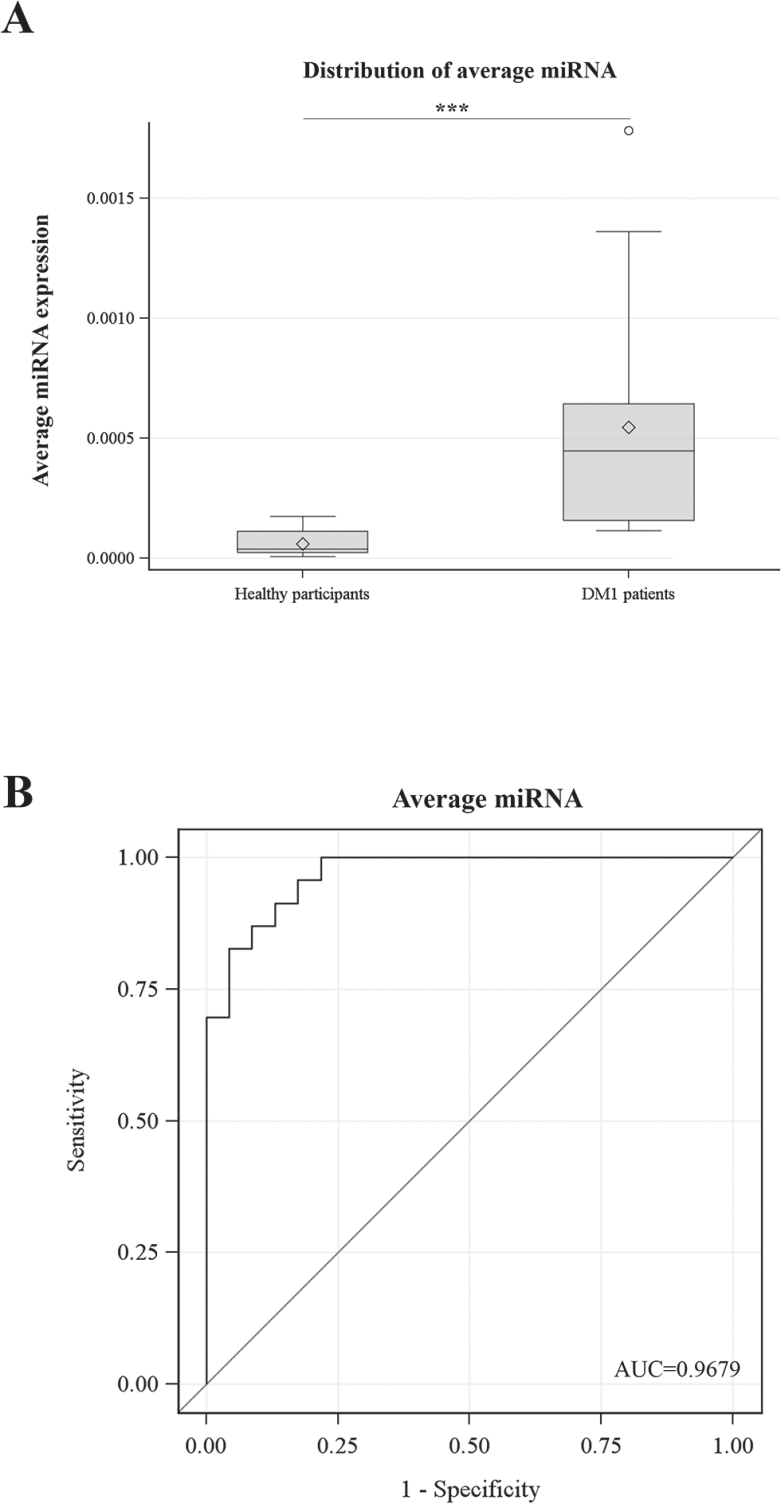
The average expression of the four miRNAs has almost the same specificity with the individual specificities of the four miRNAs. The average relative quantitation (RQ) values of the four muscle-specific miRNAs were calculated and analysed. (**A**) Distribution chart shows that the average of miRNA expression levels is elevated in DM1 patients compared to healthy participants. (**B**) ROC curve analysis shows that the average expression of the four miRNAs has almost the same specificity with the individual specificities of the four miRNAs. Area under the curve (AUC) value is presented. *** p < 0.0001.

At first, muscle-specific miRNA levels in DM1 patients were compared to levels in healthy individuals. Sera from twenty three DM1 patients and twenty three healthy participants were isolated from blood samples followed by extraction of total RNA, including miRNA. Real-Time PCR analysis was performed specific for the four muscle-specific miRNAs. The levels of miR-1, miR-133a, miR-133b and miR-206 were found to be minimal in the sera of the healthy participants (Fig 1). In contrast, miR-1, miR-133a, miR-133b and miR-206 levels were significantly higher in the sera of the DM1 patients (p<0.001) (Fig 1; Table 1). The levels of the four miRNAs were normalized to the levels of the ubiquitously expressed miR-16 which was used as an internal control [42, 43]. Means and standard deviations for miRNA levels are provided in Table 1. Receiver-operator characteristics (ROC) analysis was obtained by plotting the true positive (sensitivity) versus false positive (1-specificity). The area under the curve (AUC >0.94) (Fig 2) suggests that serum levels of miR-1, miR-133a, miR-133b and miR-206 can discriminate DM1 patients from healthy individuals extremely well. Among DM1 patients, a high correlation was observed between the four muscle-specific miRNAs (correlation coefficients: 0.84–0.94; p<0.001). Further statistical analysis was performed taking into consideration the average relative quantitation (RQ) values of the four muscle-specific miRNAs (Fig 3A). ROC curve analysis shows that a variable constructed by taking the average expression of the four miRNAs has almost the same specificity with the individual specificities of the four miRNAs (AUC = 0.97) (Fig 3B). These results show that the level of muscle-specific miRNAs in the serum of DM1 patients is higher than that of the control healthy individuals. Furthermore, statistical analysis showed that there was no correlation between myomiRs levels and age or CTG repeats size (all p-values > 0.05) (S3 Table).

**Table 1:** Statistical analysis of the muscle-specific miRNA levels in the serum of healthy participants and DM1 patients.

miRNA	Healthy participants (Mean ± sd)	DM1 patients (Mean ± sd)	p-value
miR-1	0.000043 ± 0.000036	0.00033 ± 0.00024	p<0.0001
miR-133a	0.000082 ± 0.000071	0.00063 ± 0.00053	p<0.0001
miR-133b	0.000086 ± 0.000070	0.00065 ± 0.00047	p<0.0001
miR-206	0.000031 ± 0.000031	0.00058 ± 0.00060	p<0.0001

There is an ever growing number of non-muscle-specific miRNAs found to be expressed in muscle tissue. Of these, the levels of miR-26a [49], miR-181a [50] and miR-222 [51] were investigated in the sera of DM1 patients and healthy individuals in order to verify the specificity of the presence of the myomiRs. The above miRNAs were selected, based on their high expression in muscle tissue. Moreover, their expression has not been yet related to any of the secondary diseases that affect DM1, such as type II diabetes. Real-Time PCR analysis revealed that miR-26a, miR-181a and miR-222 are present in sera isolated from both healthy individuals and DM1 patients showing no significant difference between DM1 patients and healthy individuals (S1 Fig; S4 Table). These observations support the specific presence of muscle constituents in the sera of DM1 patients.

### miR-1, miR-133a, miR-133b and miR-206 serum levels are correlated to the progress of muscle wasting

**Fig 4:**
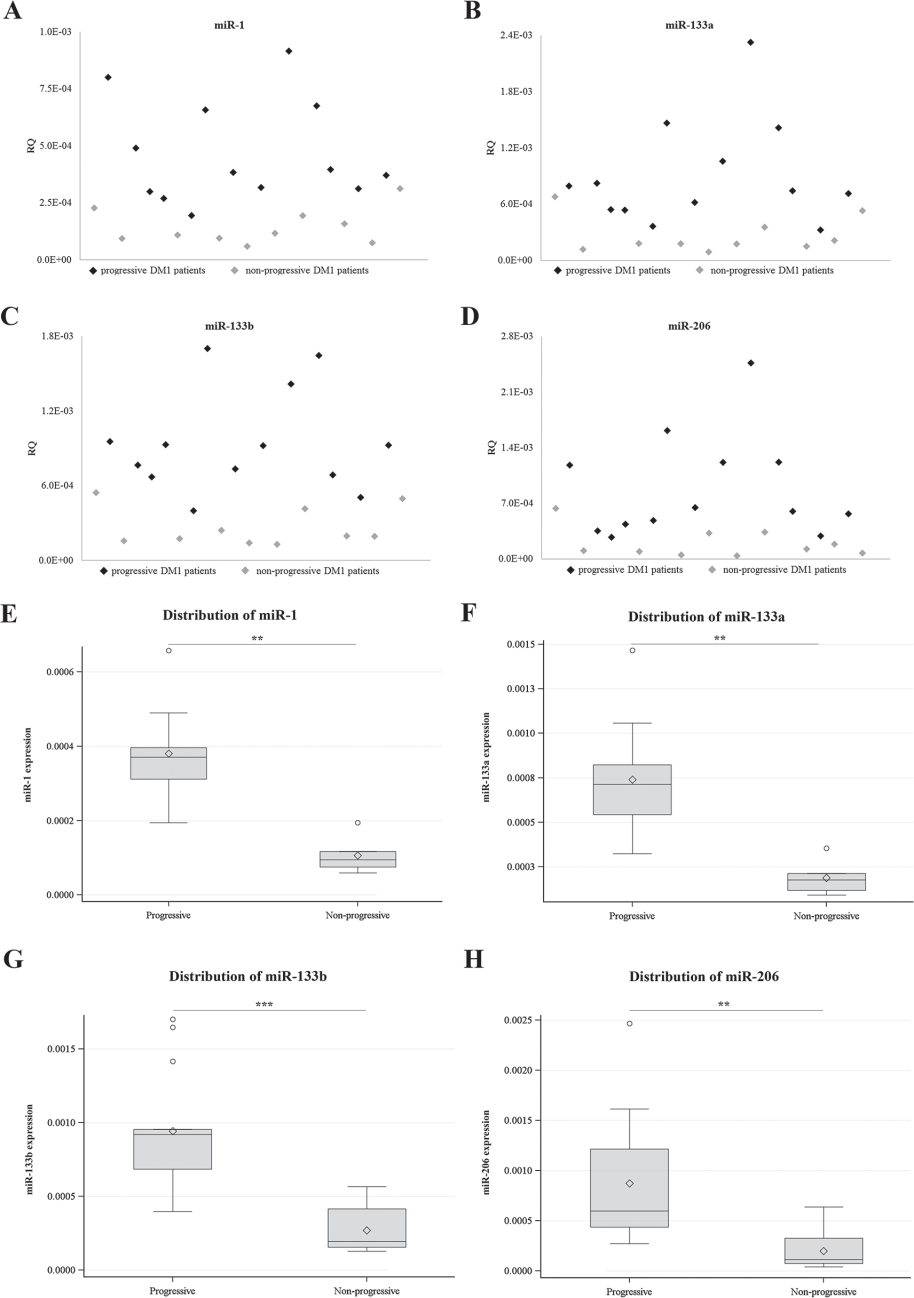
The serum levels of muscle-specific miRNAs are correlated with the progression of muscle wasting of DM1 patients. The DM1 patients were classified as progressive and non-progressive based on the progression of the muscle wasting that the patients faced at the time of blood sample collection. miR-1 (**A**), miR-133a (**B**), miR-133b (**C**) and miR-206 (**D**) serum levels were significantly higher in the progressive DM1 patients compared to the non-progressive DM1 patients (p<0.005). Distribution charts of miR-1 (**E**), miR-133a (**F**), miR-133b (**G**) and miR-206 (**H**) levels in progressive and non-progressive DM1 patients show the elevated levels of the muscle-specific miRNAs in the serum of progressive DM1 patients compared to non-progressive DM1 patients. Horizontal lines inside the boxes mark the medians. Mean expression values are marked with rhombus. *** p < 0.0001, ** p < 0.005.

**Fig 5:**
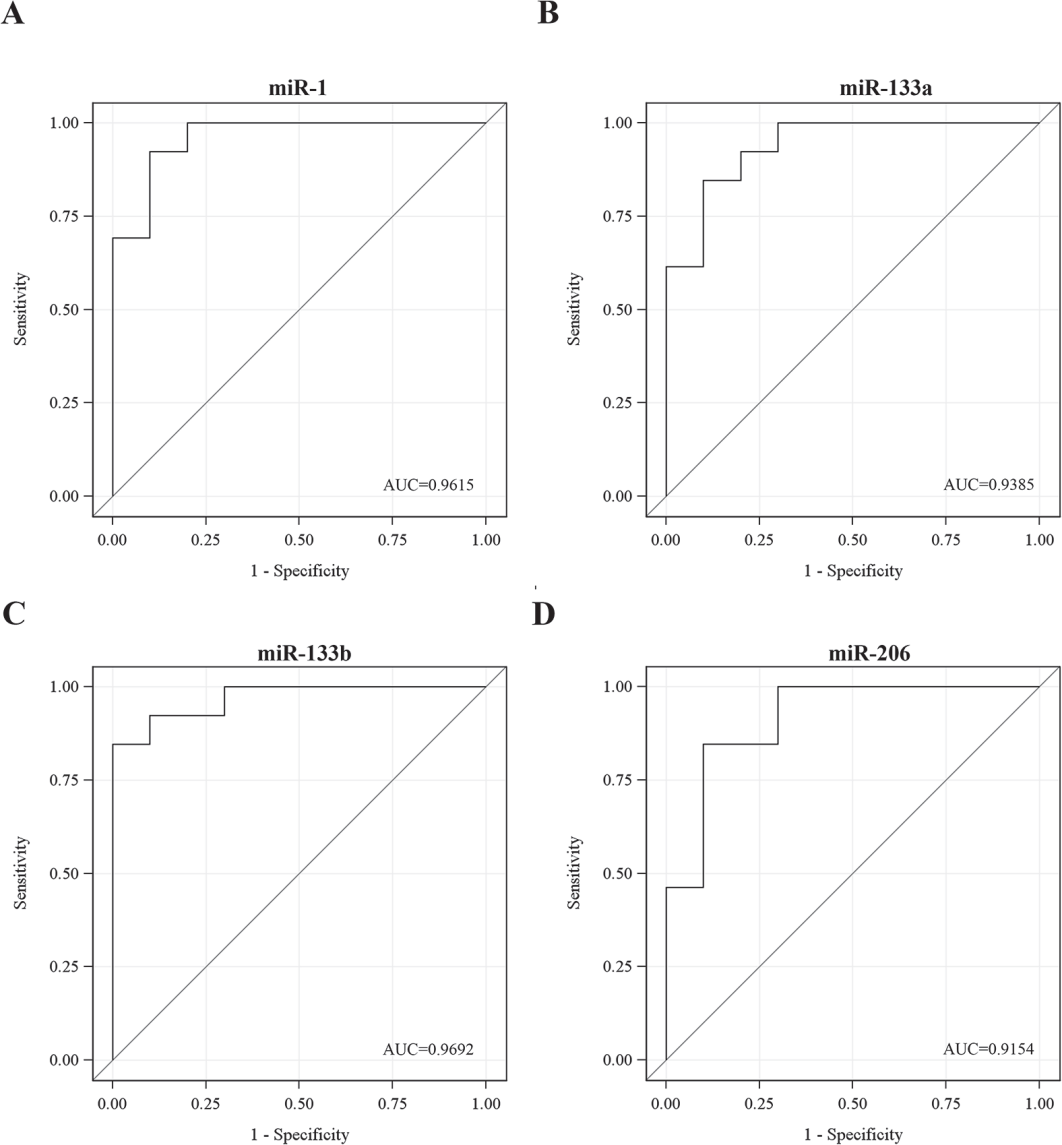
Receiver-operator characteristics (ROC) curve analyses of muscle-specific miRNAs discriminates progressive DM1 patients from non-progressive DM1 patients. ROC curve analyses using serum miR-1 (**A**), miR-133a (**B**), miR-133b (**C**) and miR-206 (**D**) for discriminating non-progressive DM1 patients from progressive DM1 patients. Area under the curve (AUC) values are presented.

**Fig 6:**
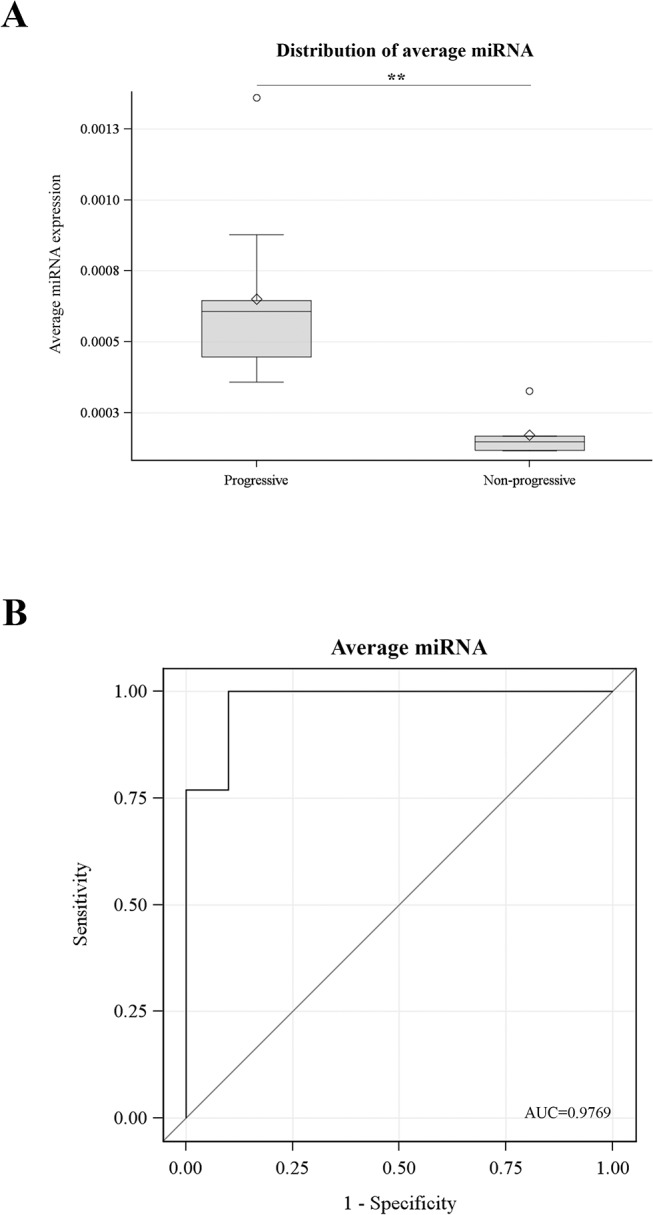
The average expression of the four miRNAs has almost the same specificity with the individual specificities of miR-1 and miR-133b. The average relative quantitation (RQ) values of the four muscle-specific miRNAs were calculated and analysed. (**A**) Distribution chart shows that the average of miRNA expression levels is elevated in progressive DM1 patients compared to non-progressive DM1 patients. (**B**) ROC curve analysis shows that the average expression of the four miRNAs has almost the same specificity with the individual specificities of miR-1 and miR-133b. Area under the curve (AUC) value is presented. ** p < 0.005.

The atrophy of muscle tissue is a major characteristic in DM1 patients. Most of the DM1 patients experience progressive muscle wasting. The severity and the rate of muscle wasting vary considerably among DM1 patients, even among members of the same family. The degradation of muscle mass however, in some cases after a period of time ceases and patients become stable. At present, the progression of muscle weakness is monitored using regular physical examinations [16]. The use of molecular biomarkers would be an additional diagnostic tool which can also be used independently in monitoring muscle wasting progress and which does not rely on prior clinical evaluation. All the patients recruited for this study were seen every four months and followed for more than a year. The neurological examination of the patients included detailed muscle power evaluation on all muscle groups (based on the Medical Research Council (MRC) scale). General haematological and biochemical examinations were performed twice yearly and cardiological assessments took place yearly. For the purposes of this study, patients without any change in the MRC scoring for the last two years were considered as disease stable (non-progressive patients). On the other hand, patients who during the last two years had scored worse in the MRC scale were considered as disease progressive patients with muscle wasting (S5 Table). Statistical analysis showed that there was no difference in age or CTG repeats size between the progressive and non-progressive DM1 patients (S6 Table). In addition, analysis showed that miR-1, miR-133a, miR-133b and miR-206 levels were significantly higher in progressive DM1 patients compared to non-progressive DM1 patients (p<0.005) (Fig 4; Table 2). Mean (standard deviation) of miRNA data are provided in Table 2. ROC analyses (using miR-1, miR-133a, miR-133b and miR-206serum levels) showed extremely high specificity in discriminating between progressive and non-progressive DM1 patients (AUC >0.91) (Fig 5). Among progressive DM1 patients, high correlation between the four muscle-specific miRNAs was observed (correlation coefficients 0.74–0.98; p<0.001). Further statistical analysis was performed taking into consideration the average RQ values of the four muscle-specific miRNAs (Fig 6A). ROC curve analysis shows that the variable constructed as the average of the four miRNAs values has almost the same specificity with the individual specificities of miR-1 and miR-133b (AUC = 0.98) (Fig 6B).

**Table 2:** Statistical analysis of muscle-specific miRNA levels in the serum of DM1 patients with non-progressive and progressive muscle wasting.

miRNA	Non-progressive DM1 patients (Mean ± sd)	Progressive DM1 patients (Mean ± sd)	p-value
miR-1	0.00015 ± 0.000084	0.00047 ± 0.00022	0.0002
miR-133a	0.00027 ± 0.00019	0.00090 ± 0.00055	0.0005
miR-133b	0.00027 ± 0.00016	0.00094 ± 0.00041	0.0002
miR-206	0.00020 ± 0.00019	0.00087 ± 0.00064	0.0009

**Fig 7:**
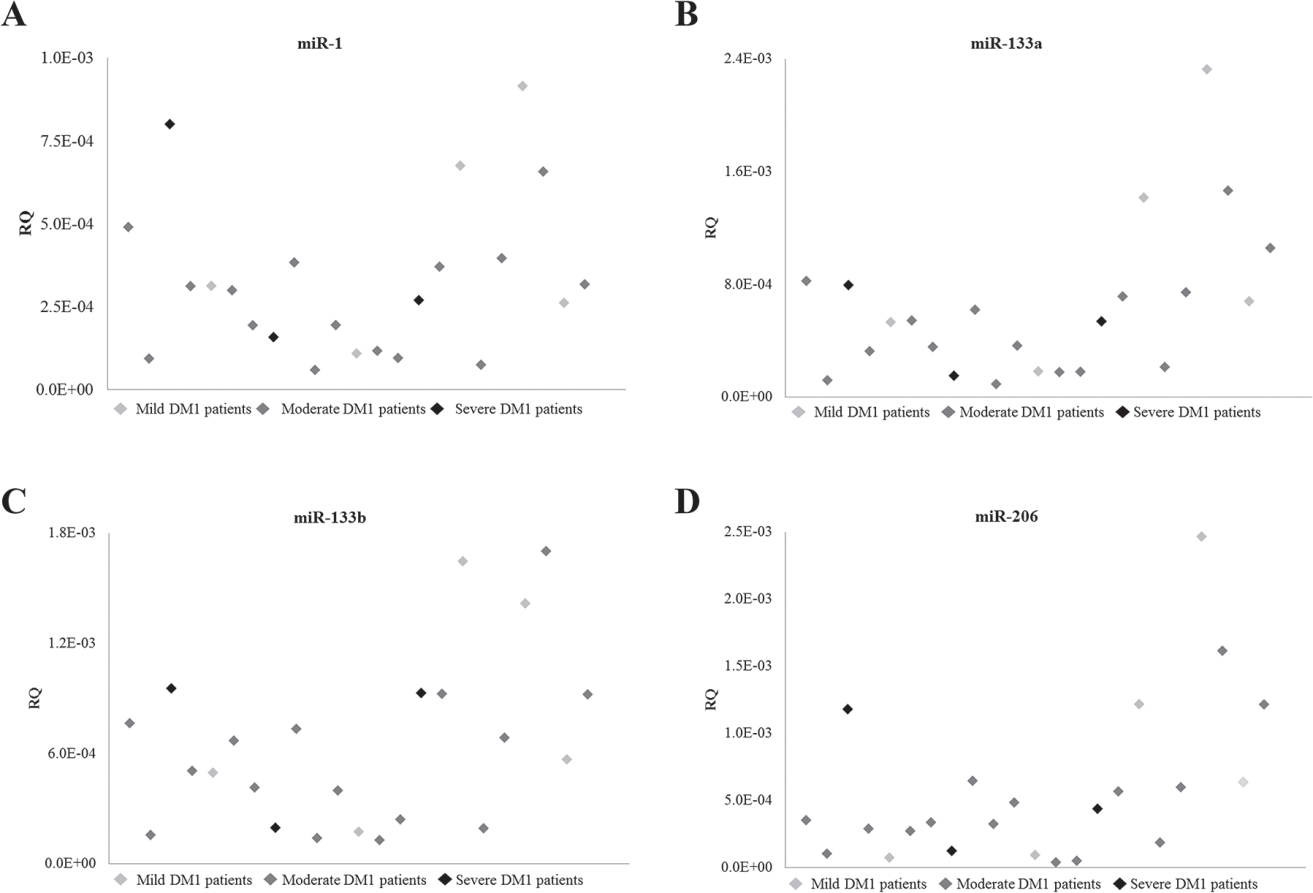
Muscle-specific miRNAs are not correlated with the severity of the disease. A comparison between the severity of the DM1 disease (mild, moderate, severe) with the levels of the muscle-specific miR-1 (**A**), miR-133a (**B**), miR-133b (**C**) and miR-206 (**D**). None of the muscle-specific miRNAs was correlated with the severity of the disease.

The relation between the levels of miR-1, miR-133a, miR-133b and miR-206 and the severity of disease was subsequently investigated. DM1 severity defines the clinical status of the patient without indicating the progression of the disease. For instance a severe patient can be stable without any progression in the disease and on the other hand a patient considered to be mild can be progressive and become moderate or severe in a short period of time. Patients were divided into three groups based on their clinical picture and the MRC scale. DM1 patients with MRC between 4 and 5 were considered mild; those with MRC score, between 3 and 4 were classified as moderate and those with MRC less than 3 were considered severe. Our results show that there is no correlation between DM1 severity and muscle-specific miRNA levels (Fig 7). In summary, our results suggest that the presence of miR-1, miR-133a, miR-133b and miR-206 in the sera of DM1 patients represents the progression of muscle wasting and not the muscle status which is determined by the clinicians at the time of the physical examination.

**Fig 8:**
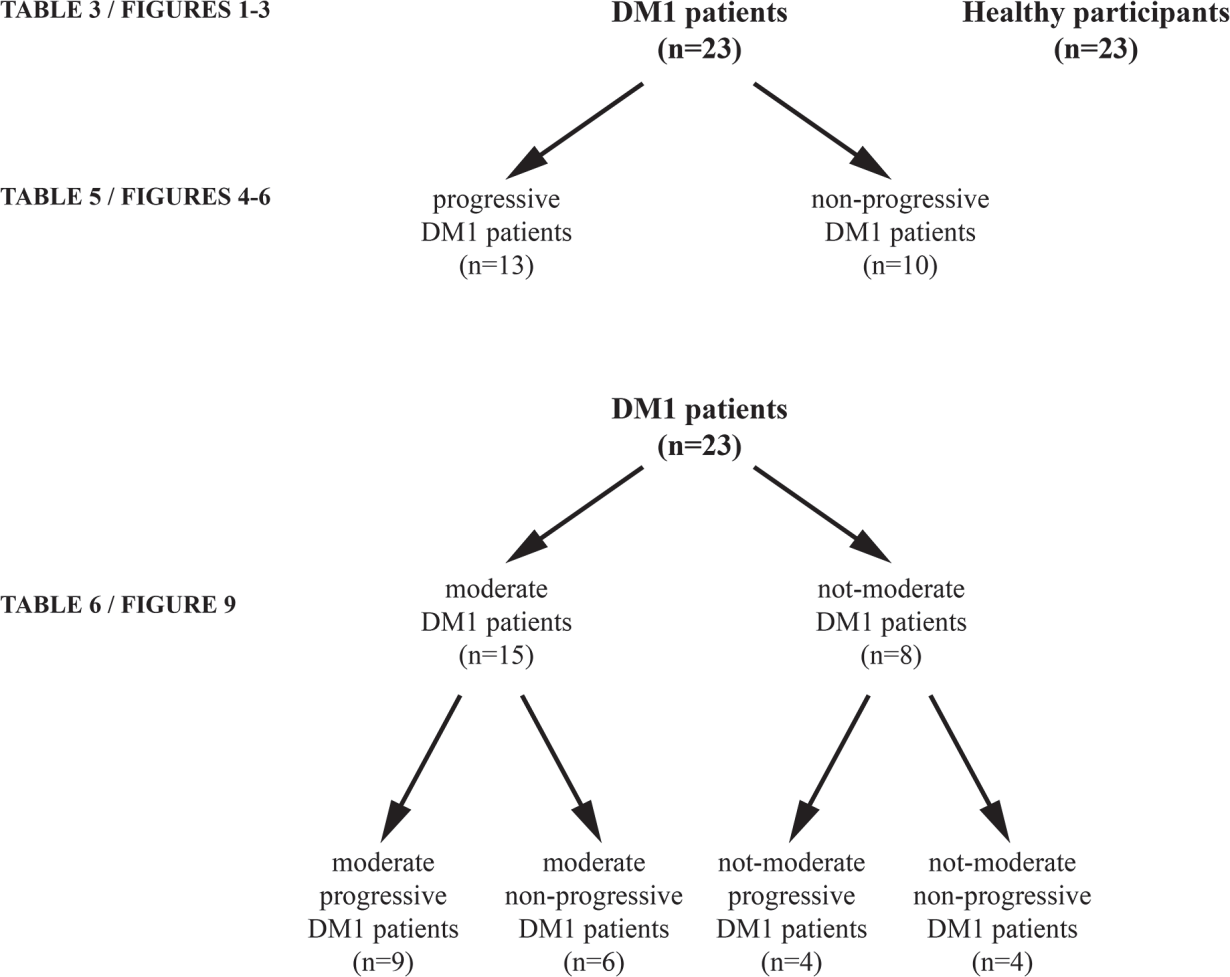
Categorization of the participants. Twenty three DM1 patients and twenty three healthy participants were enrolled to participate in the project. Thirteen DM1 patients are progressive and ten are non-progressive DM1 patients. Moderate and not-moderate patients (i.e. mild and severe DM1 patients) were also divided into progressive and non-progressive patients. Abbreviations: n: number of participants.

**Fig 9:**
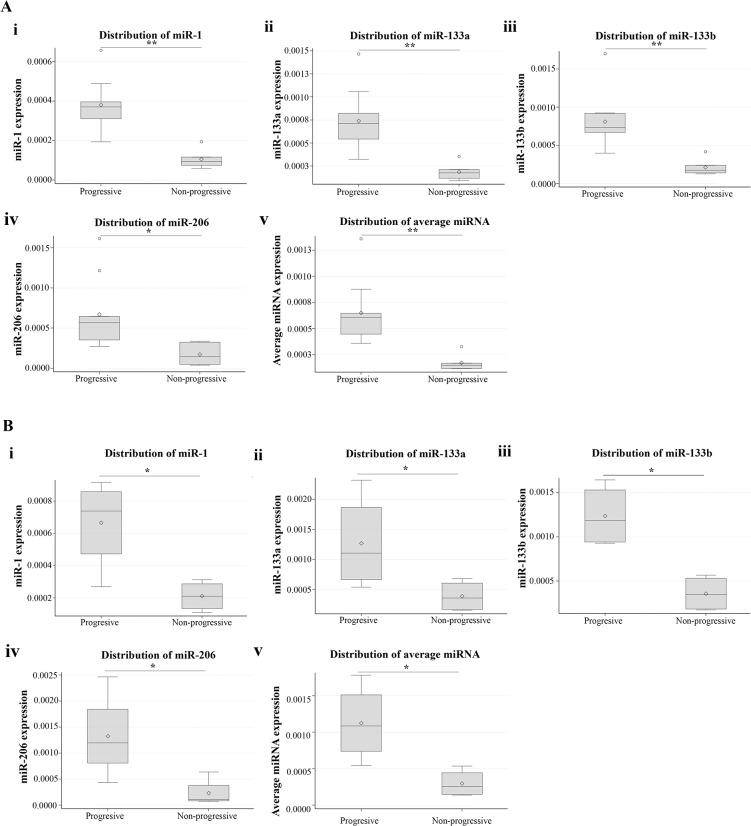
Muscle-specific miRNAs are specific for muscle wasting progress independent of the severity of the patient. DM1 patients were divided into moderate and not-moderate DM1 patients. For each of the sub-group the patients were also divided into progressive and non-progressive DM1 patients. Statistical analysis showed that in both groups, moderate DM1 patients (**A**) and not-moderate DM1 patients (**B**), the levels of the four muscle-specific miRNAs are increased in progressive DM1 patients compared to non-progressive DM1 patients. ** p < 0.005, * p < 0.05.

Our study results suggest that muscle-specific miRNAs can discriminate between progressive and non-progressive DM1 patients independently of their disease severity. To further confirm our results that suggest that muscle-specific miRNAs are specific for muscle wasting progress independent of disease severity, DM1 patients were divided into moderate and not-moderate DM1 patients (i.e. patients with mild or severe form of the disease). For each of the sub-group the patients were further divided into progressive and non-progressive DM1 patients (Fig 8). Statistical analysis showed that (a) there was no difference in age or CTG repeats size between the sub-groups (S6 Table); (b) in both moderate DM1 patients and not-moderate DM1 patients, the levels of the four muscle-specific miRNAs are increased in progressive DM1 patients compared to non-progressive DM1 patients (Fig 9). Means, standard deviations and p-values are provided in Table 3. These results confirm our results and provide additional support to our conclusion that the levels of the muscle-specific miRNAs are correlated with the progression of muscle wasting independent of disease severity.

**Table 3:** Statistical analysis of muscle-specific miRNA levels in the serum of moderate and not-moderate DM1 patients with non-progressive and progressive muscle wasting.

miRNA	Moderate DM1 patients			Not-moderate DM1 patients		
	Progressive DM1 patients (Mean ± sd)	Non-progressive DM1 patients (Mean ± sd)	p-value	Progressive DM1 patients (Mean ± sd)	Non-progressive DM1 patients (Mean ± sd)	p-value
miR-1	0.00038 ± 0.00013	0.00011 ± 0.000048	0.00040	0.00067 ± 0.00028	0.00021 ± 0.000093	0.057
miR-133a	0.00074 ± 0.00035	0.00019 ± 0.000093	0.00080	0.0013 ± 0.00080	0.00039 ± 0.00026	0.057
miR-133b	0.00081 ± 0.00038	0.00021 ± 0.00011	0.00080	0.0012 ± 0.00035	0.00036 ± 0.00020	0.029
miR-206	0.00067 ± 0.00045	0.00017 ± 0.00013	0.0048	0.0013 ± 0.00084	0.00023 ± 0.00027	0.057

## Discussion

The aim of this work was to detect and assess potential molecular biomarkers for monitoring muscle weakness and progressive atrophy in DM1 patients. Genetic material that is specifically expressed in muscle tissue was considered to be a very good candidate for characterising only the progression of muscle wasting in DM1 patients independently of the status in other tissues that might be possibly affected. Recently, miRNAs have been identified and characterised as potential biomarkers for various diseases and conditions. The presence of muscle-specific miRNAs in the blood circulation of DM1 patients was therefore assessed; the use of these miRNAs as potential biomarkers of muscle wasting was evaluated.

The levels of the four muscle-specific miRNAs, miR-1, miR-133a, miR-133b and miR-206, were initially detected in the sera isolated from DM1 patients and compared to healthy participants. Following RNA analysis, muscle-specific miRNA levels were found significantly increased in the serum of DM1 patients, as compared to levels in normal participants which were found to be minimal. Similar reports were published showing this association in the serum and/or plasma with other diseases (DMD and congenital muscular dystrophy type 1A (MDC1A)) [52, 53]. Very recently, nine miRNAs were found to be altered in plasma samples of DM1 patients compared to controls. One of the miRNA evaluated, miR-133a is muscle-specific [47]. All these results point to the conclusion that muscle-specific miRNAs may be considered valid biomarkers for muscular dystrophy.

Muscle weakness and wasting is a primary characteristic of DM1 patients and the main cause of their disability. Muscle wasting generally worsens over time, but the rate of deterioration varies between patients. Markers for the characterisation of muscle wasting progression would therefore be of value for DM1 patients. The levels of the four muscle-specific miRNA were found to be correlated with the progression of muscle weakness and wasting in DM1 patients. Specifically, miR-1, miR-133a, miR-133b and miR-206 levels were found to be significantly higher in progressive DM1 patients, compared to non-progressive DM1 patients. This is the first report showing evidence of a correlation between miRNA levels in blood and muscle wasting progress in DM1 patients. These results imply that these muscle-specific miRNAs presumably leak from the degraded muscle tissue during muscle wasting and enter the blood circulation of the patient. The specificity of the muscle-specific miRNAs leakage was further supported by the observation that other non-muscle-specific miRNAs were not found to be elevated in the blood circulation of DM1 patients when they were compared to the controls. The results presented here propose also that the levels of the four miRNAs are reduced when muscle wasting is stabilized, independent of disease severity. miRNAs are known to be relatively stable in the blood circulation and appear resistant to ribonucleases (RNase) which are present in the blood, however the half time of the miRNAs within blood circulation remains unknown [38, 54]. Recent studies showed that the encapsulation of the miRNAs into membrane-vesicles provide a general protection for the extracellular circulating miRNAs. Specifically, it has been found that microvesicles and exosomes that derive from the cells contain miRNAs thus protecting them from the RNAses activities [55, 56]. In other recent studies, it has been demonstrated that the extracellular circulating miRNAs are not encapsulated into vesicles and are associated with Ago1 and/or Ago2 proteins both in blood plasma/serum and cell culture media [57–59]. Ago1 and Ago2 are the proteins that miRNAs are naturally associated within the cells and are part of the RNA-induced silencing complex. The stability of Ago proteins in protease rich environment explained the resistance of Ago-bound miRNAs in nucleases that exist in biological fluids [57–59]. Some miRNAs were also identified to be protected by associating with the high-density lipoprotein (HDL) and exist in the blood circulation [60, 61]. The decrease in the serum levels of muscle-specific miRNAs in non-progressive DM1 patients suggests that the miRNAs present in the blood circulation are probably degraded after a period of time by which muscle wasting had ceased.

The increase in the serum levels of miR-1, miR-133a, miR-133b and miR-206 observed in DM1 patients was not correlated with disease severity. It should be noted however that a recent report showed that muscle-specific miRNA levels in serum of DMD patients correlated with the severity of the disease [43]. DMD is an inheritable lethal childhood’s disease and patients show signs of muscle weakness as early as the age of two with high progressive rate. DM1 however, is a highly variable disease. The age of onset and the rate of disease progression highly vary among the patients and it cannot be predicted. In DM1 patients, disease severity underpins the muscle situation at the time of examination and it can be easily assessed by physical examination. Disease progression and muscle wasting cannot however be evaluated at the time of the examination. For instance, a patient may be stable with the mild or the moderate or the severe form of the disease or it can be progressive with mild or moderate or severe form of the disease. The severity of disease can be easily assessed the physical examination. Currently, the progression of muscle wasting is monitored through the use of regular physical examinations [16]. The use of molecular biomarkers would be an additional diagnostic tool which can be also used independently in monitoring muscle wasting progression and which does not rely on previous clinical evaluations.

Recent research has shown that miRNAs may be useful tools for the screening of several diseases, including cancers, and injuries [62–64]. In the present work, we show that the four muscle-specific miRNAs, miR-1, miR-133a, miR-133b and miR-206, are elevated in the serum of DM1 patients, compared to control participants. Muscle tissue wasting is a major characteristic of DM1 patients. Most of the DM1 patients experience progressive muscle atrophy. However, the degradation of muscle mass in some cases after a period of time, ceases and patients become stable independent of their disease severity. We show that the levels of the four muscle-specific miRNAs are significantly elevated in DM1 patients with progressive muscle wasting compared to patients with non-progressive muscle wasting. This observation suggests that these miRNAs are released from the degraded muscle tissue into the blood circulation. These results propose that muscle-specific miRNAs can be used as potential serum-based molecular biomarkers for monitoring the progress of muscle wasting in DM1 patients. The high specificity of these miRNAs in the serum of DM1 patients as shown by ROC analysis implies that miRNA values can be used to generate a binary molecular biomarker in order to differentiate between progressive and non-progressive DM1 patients. The presence of such biomarkers could be used as a tool for the follow-up of muscle wasting in DM1 as well as for other muscle diseases causing muscles’ atrophy. Although there are several studies that support the idea that miRNAs can potentially be used as clinical non-invasive biomarkers for various diseases, an understanding of the ontology of serum miRNA would be important for a precise clinical interpretation which could in turn render them more reliable biomarkers in clinical practice. Moreover, a succinct determination of the nature of the four muscle-specific miRNAs in the blood circulation of DM1 patients could provide information regarding the pathology of muscle in DM1 and the miRNA release mechanisms. The development of a reliable biomarker for monitoring and characterizing muscle wasting in DM1 patients will give the opportunity to the clinicians to have a regular and better monitoring of patient progress. Moreover, the detection of these miRNAs can help towards a better understanding of the efficacy of current drugs and the evaluation of future clinical trials.

## Supporting Information

S1 Fig The presence of the muscle-derived miR-26a (A), miR-181a (B) and miR-222 (C) was not altered in the serum of DM1 patients compared to the serum of healthy participants. (TIF)

S1 Table Characteristics of healthy participants and DM1 patients. (DOCX)

S2 Table Characteristics and clinical investigation of DM1 patients. (XLSX)

S3 Table Comparison between age and average CTG repeats size and miRNAs levels. (DOCX)

S4 Table Statistical analysis of the miRNAs that are highly expressed in muscle tissue in the serum of healthy participants and DM1 patients. (DOCX)

S5 Table Muscle power examination changes observed in DM1 patients (years 2010–2014) based on MRC scale (1–5). (DOCX)

S6 Table Correlation between miRNAs levels and age or CTG repeats size in progressive and non-progressive moderate DM1 patients. (DOCX)
