# Supplementary material for: Elevated Muscle-Specific miRNAs in Serum of Myotonic Dystrophy Patients Relate to Muscle Disease Progress
Source: PLoS One. 2015 Apr 27;10(4):e0125341. doi: 10.1371/journal.pone.0125341 (PMC4411125; doi:10.1371/journal.pone.0125341)
Supplement: S1 Table — (DOCX) [file pone.0125341.s002.docx]

| **Characteristic** | **Healthy participants**  **n=23 (50%)** | **DM1 patients**  **n=23 (50%)** |
| --- | --- | --- |
| **Sex** | | |
| Male (%) | 14 (61%) | 15 (65%) |
| Female (%) | 9 (39%) | 8 (35%) |
| **Age of individual at time of sample collection** | | |
| Median (range) | 40 (26 – 59) | 41 (24 – 63) |

**S1 Table. Characteristics of healthy participants and DM1 patients.**
