# Supplementary material for: Elevated Muscle-Specific miRNAs in Serum of Myotonic Dystrophy Patients Relate to Muscle Disease Progress
Source: PLoS One. 2015 Apr 27;10(4):e0125341. doi: 10.1371/journal.pone.0125341 (PMC4411125; doi:10.1371/journal.pone.0125341)
Supplement: S3 Table — (DOCX) [file pone.0125341.s004.docx]

**S3 Table. Comparison between age and average CTG repeats size and miRNAs levels.**

| **DM1 patients (n=23)** | | | | | |
| --- | --- | --- | --- | --- | --- |
|  | **Mean (± sd)** | **miR-1**  p-value | **miR-133a**  p-value | **miR-133b**  p-value | **miR-206**  p-value |
| **Age** | 40.39 (±10.05) | 0.98 | 0.85 | 0.54 | 0.86 |
| **Average CTG repeats size** | 582.17 (±273.62) | 0.51 | 0.35 | 0.33 | 0.54 |
