# Supplementary material for: Elevated Muscle-Specific miRNAs in Serum of Myotonic Dystrophy Patients Relate to Muscle Disease Progress
Source: PLoS One. 2015 Apr 27;10(4):e0125341. doi: 10.1371/journal.pone.0125341 (PMC4411125; doi:10.1371/journal.pone.0125341)
Supplement: S4 Table — (DOCX) [file pone.0125341.s005.docx]

**S4 Table. Statistical analysis of the miRNAs that are highly expressed in muscle tissue in the serum of healthy participants and DM1 patients.**

| **miRNA** | **Healthy participants**  **(Mean ± sd)** | **DM1 patients**  **(Mean ± sd)** | **p-value** |
| --- | --- | --- | --- |
| miR-26a | 0.0170875 ± 0.0049771 | 0.0219744 ± 0.0111005 | 0.3918 |
| miR-181a | 0.0023719 ± 0.00097941 | 0.0026717 ± 0.0024546 | 0.5309 |
| miR-222 | 0.0054094 ± 0.0016918 | 0.0065128 ± 0.0029962 | 0.5691 |

Due to the small sample size, non-parametric techniques were used to make comparisons between the groups_._
