# Supplementary material for: Elevated Muscle-Specific miRNAs in Serum of Myotonic Dystrophy Patients Relate to Muscle Disease Progress
Source: PLoS One. 2015 Apr 27;10(4):e0125341. doi: 10.1371/journal.pone.0125341 (PMC4411125; doi:10.1371/journal.pone.0125341)
Supplement: S5 Table — (DOCX) [file pone.0125341.s006.docx]

**S5 Table. Muscle power examination changes observed in DM1 patients (years 2010-2014) based on MRC scale (1-5).**

| **DM1 patients** | **Movement description** | | | |
| --- | --- | --- | --- | --- |
|  | **2010/2011** | **2011/2012** | **2012/2013** | **2013/2014** |
| **Patient 1** | Hip flexion 5/5R 5/5L  Knee flexion 4/5R 5/5L  Knee extension 5/5R 5/5L | Hip flexion 4/5R 4/5L  Knee flexion 4^-^/5R 4/5L  Knee extension 4/5R 4/5L |  |  |
| **Patient 2** | Wrist extension 4/5R 4/5L  Finger extension 3/5R 3/5L  Finger abduction 3/5R 3/5L  Hip flexion 4^-^/5R 4^+^/5L  Knee extension 4^+^/5R 4^+^/5L  Foot dorsiflexion 3/5R 3/5L  Plantar flexion 3/5R 3/5L | Wrist extension 4/5R 4/5L  Finger extension 3/5R 3/5L  Finger abduction 3/5R 3/5L  Hip flexion 4^-^/5R 4^+^/5L  Knee extension 4^+^/5R 4^+^/5L  Foot dorsiflexion 3/5R 3/5L  Plantar flexion 3/5R 3/5L |  |  |
| **Patient 3** | Arm abduction 5/5R 5/5L  Arm flexion 5/5R 5/5L  Wrist extension 4/5R 4/5L  Foot dorsiflexion 4/5R 4/5L | Arm abduction 4/5R 4/5L  Arm flexion 4/5R 4/5L  Wrist extension 3/5R 3/5L  Foot dorsiflexion 3/5R 3/5L |  |  |
| **Patient 4** | Wrist extension 4/5R 4/5L  Finger extension 4^-^/5R 4^-^/5L  Finger abduction 4^-^/5R 4^-^/5L  Foot dorsiflexion 3^+^/5R 4^-^/5L | Wrist extension 4/5R 4/5L  Finger extension 4^-^/5R 4^-^/5L  Finger abduction 4^-^/5R 4^-^/5L  Foot dorsiflexion 3^+^/5R 4^-^/5L |  |  |
| **Patient 5** | Head flexion 4/5R 4/5L  Wrist extension 4^-^/5R 4^-^/5L  Finger extension 3/5R 3/5L  Finger abduction 3/5R 3/5L  Hip flexion 4^-^/5R 4^-^/5L  Knee extension 2/5R 2/5L  Knee flexion 4^-^/5R 4^-^/5L  Foot dorsiflexion 0/5R 0/5L  Plantar flexion 0/5R 0/5L | Head flexion 4/5R 4/5L  Wrist extension 4^-^/5R 4^-^/5L  Finger extension 3/5R 3/5L  Finger abduction 3/5R 3/5L  Hip flexion 4^-^/5R 4^-^/5L  Knee extension 2/5R 2/5L  Knee flexion 4^-^/5R 4^-^/5L  Foot dorsiflexion 0/5R 0/5L  Plantar flexion 0/5R 0/5L |  |  |
| **Patient 6** | Elbow extension 5/5R 4/5L  Foot dorsiflexion 5/5R 5/5L | Elbow extension 4/5R 4/5L  Foot dorsiflexion 4/5R 5/5L |  |  |
| **Patient 7** | Finger extension 4/5R 4/5L  Finger abduction 4/5R 4/5L | Finger extension 4/5R 4/5L  Finger abduction 4/5R 4/5L |  |  |
| **Patient 8** | Wrist extension 5/5R 5/5L  Foot dorsiflexion 5/5R 5/5L | Wrist extension 4/5R 4/5L  Foot dorsiflexion 4^-^/5R 4/5L |  |  |
| **Patient 9** | Head flexion 4^+^/5R 4^+^/5L  Finger extension 4^+^/5R 4^+^/5L  Finger abduction 4/5R 4/5L  Foot dorsiflexion 4^+^/5R 4^+^/5L | Head flexion 4^+^/5R 4^+^/5L  Finger extension 4^+^/5R 4^+^/5L  Finger abduction 4/5R 4/5L  Foot dorsiflexion 4^+^/5R 4^+^/5L |  |  |
| **Patient 10** | Finger extension 4/5R 4/5L  Finger abduction 4/5R 4/5L | Finger extension 4/5R 4/5L  Finger abduction 4/5R 4/5L |  |  |
| **Patient 11** | Hip flexion 5/5R 5/5L  Knee flexion 5/5R 5/5L | Hip flexion 4/5R 4^+^/5L  Knee flexion 4^+^/5R 5/5L |  |  |
| **Patient 12** | Wrist extension 3/5R 3/5L  Finger extension 3/5R 3/5L  Finger abduction 3/5R 3/5L | Wrist extension 3/5R 3/5L  Finger extension 3/5R 3/5L  Finger abduction 3/5R 3/5L |  |  |
| **Patient 13** | Arm flexion 4/5R 4/5L  Wrist extension 4/5R 4/5L  Foot dorsiflexion 5/5R 5/5L | Arm flexion 3^+^/5R 3^+^/5L  Wrist extension 3^+^/5R 3^+^/5L  Foot dorsiflexion 4^+^/5R 4^+^/5L |  |  |
| **Patient 14** | Finger extension 4/5R 4/5L  Finger abduction 4/5R 4/5L  Foot dorsiflexion 4/5R 4/5L  Plantar flexion 4/5R 4/5L | Finger extension 4/5R 4/5L  Finger abduction 4/5R 4/5L  Foot dorsiflexion 4/5R 4/5L  Plantar flexion 4/5R 4/5L |  |  |
| **Patient 15** | Hip flexion 5/5R 5/5L  Knee flexion 5/5R 5/5L  Knee extension 5/5R 5/5L  Arm abduction 5/5R 5/5L | Hip flexion 4/5R 4/5L  Knee flexion 5/5R 4^-^/5L  Knee extension 4^+^/5R 4^+^/5L  Arm abduction 5/5R 4/5L |  |  |
| **Patient 16** |  |  | Arm flexion 4/5R 4/5L | Arm flexion 3^+^/5R 3^+^/5L |
| **Patient 17** |  |  | Foot dorsiflexion 5/5R 5/5L | Foot dorsiflexion 4^+^/5R 4^+^/5L |
| **Patient 18** |  |  | Hip flexion 5/5R 5/5L | Hip flexion 4/5R 4/5L |
| **Patient 19** |  |  | Wrist extension 4^+^/5R 4^+^/5L  Hip flexion 5/5R 5/5L  Foot dorsiflexion 5/5R 5/5L  Arm flexion 5/5R 5/5L | Wrist extension 4^+^/5R 4^+^/5L  Hip flexion 5/5R 5/5L  Foot dorsiflexion 5/5R 5/5L  Arm flexion 5/5R 5/5L |
| **Patient 20** |  |  | Wrist extension 5/5R 5/5L  Hip flexion 5/5R 5/5L  Foot dorsiflexion 5/5R 5/5L  Arm flexion 5/5R 5/5L | Wrist extension 5/5R 5/5L  Hip flexion 5/5R 5/5L  Foot dorsiflexion 5/5R 5/5L  Arm flexion 5/5R 5/5L |
| **Patient 21** |  |  | Foot dorsiflexion 5/5R 5/5L | Foot dorsiflexion 4/5R 4/5L |
| **Patient 22** |  |  | Wrist extension 4/5R 4/5L | Wrist extension 4^-^/5R 4^-^/5L |
| **Patient 23** |  |  | Wrist extension 5/5R 5/5L | Wrist extension 4/5R 4/5L |

Abbreviations: R: Right; L: Left
