# Supplementary material for: Elevated Muscle-Specific miRNAs in Serum of Myotonic Dystrophy Patients Relate to Muscle Disease Progress
Source: PLoS One. 2015 Apr 27;10(4):e0125341. doi: 10.1371/journal.pone.0125341 (PMC4411125; doi:10.1371/journal.pone.0125341)
Supplement: S6 Table — (DOCX) [file pone.0125341.s007.docx]

**S6 Table. Correlation between miRNAs levels and age or CTG repeats size in progressive and non-progressive moderate DM1 patients.**

|  |  | **AGE** | | | **Average CTG repeats** | | |
| --- | --- | --- | --- | --- | --- | --- | --- |
|  |  | **Moderate DM1 patients** | **Not-moderate DM1 patients** | **All DM1 patients** | **Moderate DM1 patients** | **Not-moderate DM1 patients** | **All DM1 patients** |
| **Progressive DM1 patients** | **Mean (±sd)** | 36.44 (±6.80) | 41.25 (±8.92) | 37.92 (±7.49) | 531.67  (±222.74) | 500.00  (±234.52) | 521.92 (±216.92) |
|  | **Number of patients** | 9 | 4 | 13 | 9 | 4 | 13 |
| **Non-progressive DM1 patients** | **Mean (±sd)** | 38.17 (±9.62) | 51.75 (±12.42) | 43.60 (±12.33) | 692.50  (±332.17) | 612.50  (±368.27) | 660.50 (±328.96) |
|  | **Number of patients** | 6 | 4 | 10 | 6 | 4 | 10 |
|  | **p-value** | 0.67 | 0.20 | 0.25 | 0.34 | 0.77 | 0.27 |

p-values relate to the non-parametric comparisons of progressive and non-progressive DM1 patients
